# Genetic links between post-reproductive lifespan and family size in Framingham

**DOI:** 10.1093/emph/eot013

**Published:** 2013-06-25

**Authors:** Xiaofei Wang, Sean G. Byars, Stephen C. Stearns

**Affiliations:** ^1^Department of Statistics, Yale University, New Haven, CT 06520-8102, USA, ^2^Department of Biology, Copenhagen University, Universitetsparken 15, 2100 Copenhagen, Denmark and ^3^Department of Ecology and Evolutionary Biology, Yale University, New Haven, CT 06520-8102, USA

**Keywords:** genome-wide association study, longevity, trade-off, family size

## Abstract

**Background and objectives:** Is there a trade-off between children ever born (CEB) and post-reproductive lifespan in humans? Here, we report a comprehensive analysis of reproductive trade-offs in the Framingham Heart Study (FHS) dataset using phenotypic and genotypic correlations and a genome-wide association study (GWAS) to look for single-nucleotide polymorphisms (SNPs) that are related to the association between CEB and lifespan.

**Methodology:** We calculated the phenotypic and genetic correlations of lifespan with CEB for men and women in the Framingham dataset, and then performed a GWAS to search for SNPs that might affect the relationship between post-reproductive lifespan and CEB.

**Results:** We found significant negative phenotypic correlations between CEB and lifespan in both women (*r*_P_ = −0.133, *P* < 0.001) and men (*r*_P_ = −0. 079, *P* = 0.036). The genetic correlation was large, highly significant and strongly negative in women (*r*_G_ = −0.877, *P* = 0.009) in a model without covariates, but not in men (*P* = 0.777). The GWAS identified five SNPs associated with the relationship between CEB and post-reproductive lifespan in women; some are near genes that have been linked to cancer. None were identified in men.

**Conclusions and implications:** We identified several SNPs for which the relationship between CEB and post-reproductive lifespan differs by genotype in women in the FHS who were born between 1889 and 1958. That result was not robust to changes in the sample. Further studies on larger samples are needed to validate the antagonistic pleiotropy of these genes.

## BACKGROUND AND OBJECTIVES

Both the theory of life-history evolution and the evolutionary theory of aging assume a trade-off between reproduction and survival: a cost of reproduction paid in lifespan [1–4]. Although well documented in model organisms, the existence of this trade-off in humans has been controversial (e.g. [5]). Negative [6–11], positive [12–17], U-shaped [18–20] and mixed or insignificant [21–27] relationships between completed family size and lifespan have all been found. Some results have been criticized on statistical grounds; some authors doubt that the trade-off exists at all (e.g. [28–32]). Two papers suggest that the cost is only expressed in women of low social class or nutritional status; a similar effect has been found in model organisms [5, 21, 27].

Although most of the attempts to measure the trade-off in humans are based on phenotypic correlations, the standard of evidence for the existence of a trade-off in evolutionary analyses of model organisms is a negative genetic correlation demonstrated as a correlated response to selection (e.g. [5, 33]). Such experiments reveal genetic relationships often hidden by phenotypic plasticity. This standard cannot be met in humans, where experimental evolution is not possible.

Two other types of genetic evidence, however, are available in humans. First, genetic correlations can be measured with pedigree analysis using methods developed for animal breeding. Using such methods, Gögele *et al.* [34] found a significantly ‘positive’ genetic correlation between completed family size and lifespan in a sample of more than 5100 men and women who lived between 1658 and 1907 in South Tyrol, Italy.

Second, genome-wide association studies (GWAS) can be done on populations where both the relevant traits and the single-nucleotide polymorphisms (SNPs) have been measured. In a GWAS done on more than 3500 women from Rotterdam, Kuningas *et al.* [35] found four chromosomal regions that influenced completed family size; none of them appeared also to affect lifespan.

The aims of this analysis of men and women in the Framingham Heart Study (FHS) were to add to the genetic information on reproductive trade-offs in humans by (i) first measuring the phenotypic correlation of lifespan with children ever born (CEB), (ii) second estimating the genetic correlation of lifespan with CEB and (iii) performing a GWAS to search for SNPs with effects on the relationship of lifespan to CEB. We found significantly negative phenotypic and genetic correlations between post-reproductive lifespan and CEB in women. We also found five chromosomal regions mediating the trade-off that were genome-wide significant in several statistical models but not when we added smoking as a covariate. Some of the genes in those five regions are associated with increased risk of cancer.

## METHODOLOGY

### The Framingham Heart Study

Initiated in 1948 in the town of Framingham (MA), the FHS includes three generations of participants that continue to be measured. Beginning with 5209 men and women initially enrolled in the original-cohort, the study added 5124 offspring-cohort participants in 1971 that were mostly offspring of the original-cohort. In 2002, a third-cohort was added consisting of offspring of the second cohort. Original-cohort participants have been examined every 2 years (28 exams in total to date), the offspring-cohort every 4 years (eight exams in total). Participants are mostly of European ancestry (20% UK, 40% Ireland, 10% Italy and 10% Quebec). Data were de-identified by the FHS. Data-use and human subjects’ approval were obtained from the National Institutes of Health (dbGaP) and the Yale Institutional Review Board.

### Phenotypic correlations

Our sample included men and women who were born between the 1890s and the 1950s, except for age at menarche where the available sample was much smaller (i.e. 1923–56). Cox regression was used to calculate risk of death depending on age at first birth (*n*_men_ = 2579; *n*_women_ = 2193), CEB (*n*_men_ = 3833; *n*_women_ = 3658), and age at menarche (*n* = 1355) and menopause (*n* = 2415) in women. In each regression, potentially confounding effects in lifespan were controlled by including education, country of origin and smoking status. To test for potential nonlinear effects, a separate regression was run with a quadratic term included for the main predictor traits. If quadratic terms were significant, this was explored further by examining the Cox regression model (from the survival library in R) using penalized splines (with 4 df) [36, 37].

The Cox proportional hazards model is a standard tool for survival analysis, in which the log of the hazard function *h*(*t*) is assumed to be a linear combination of the covariates. Specifically, for a model containing *p* covariates 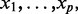 the fitted model takes the form of

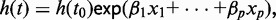

where 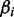 is the coefficient fit to covariate 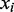 and 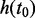 is the unknown baseline hazard function. Equivalently, this equation can be expressed as

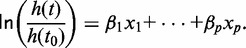



Note that FHS reports CEB as a value from ‘0’ to ‘5’, where ‘5’ indicates having had five or more children. Several variables were pre-adjusted for age and year measured. For body mass index (BMI), systolic blood pressure (SBP) and total cholesterol, age and year effects were removed by taking residuals of each trait against age (measures between 20 and 60 years old) and year measured using a generalized additive model (locally weighted scatterplot smoothing, LOESS). All residuals for a subject were then averaged to obtain an average residual for each trait, which were then used for modelling. As demonstrated previously, the surface of the generalized additive model can be accurately estimated due to the large number of trait measurements [38].

Our initial sample included 4123 women for whom data on age at death, CEB, education level, smoking history, estrogen use and BMI were available. We then removed 941 women who were born in or after 1941, a period when the correlation between lifespan and CEB was weaker, possibly because of the improvement of health care after World War II. We did so because to have a chance of detecting any significantly correlated SNPs in the GWAS, we needed to focus on a period where the phenotypic correlation is relatively strong. Nineteen women who died before the age of 50 years were also excluded, because their CEB records might represent incomplete observations. Because we excluded women who died before the age of 50 years, we are specifically studying the relationship of CEB to post-reproductive mortality. Of the remaining 3163 women, keeping only those who had genotype data reduced our sample size to 1810. We required this sample to have associated genotype data because we later used the same sample for the GWAS. Note that our phenotypic analysis used the year 1919 as a cut-off because the yearly ratio of individuals alive to individuals deceased increased to about 50% in 1919, and continued to rise thereafter.

For illustrative purposes, we also ran a multiple linear regression on a smaller sample for women, including only the deceased subjects who were born prior to 1919 (*n* = 680) out of a total of 1810 who satisfied specific criteria outlined above.

We similarly ran a regression model on a smaller sample of men who have died (*n* = 712) out of a total of 1474 men satisfying similar criteria.

### Genetic correlations and heritabilities

We estimated heritabilities and genetic correlations for traits from pedigrees using a mixed effects restricted maximum likelihood (REML) model in ASReml version 3.0 [39]. We considered models in which there were no covariates as well as adjusted models where phenotypic variation was partitioned into additive genetic, residual variance and a single random effect (maternal ID, paternal ID or education level). To be consistent with the phenotypic correlation models, we also considered models in which fixed effects (smoking status and country of origin) and both random effects for maternal ID and education level were included. Sex was not included as a fixed effect as male and female estimates were obtained separately. Smoking status (0/1, non-smoker/smoker) and country of origin (0/1, US born/foreign born) were coded as binary variables. Education described number of years completed, with missing values coded as 8 years (the minimum). Maternal variance components ranged from 0.0 (age at first birth) to 0.12 ± 0.04 (lifespan) and 0.0 (age at first birth) to 0.20 ± 0.03 (lifespan) for female and male analyses, respectively. Education variance components ranged from 0.0 (age at menarche) to 0.06 ± 0.03 (CEB) and 0.0 (age at first birth) to 0.014 ± 0.009 (CEB) for female and male analyses, respectively. The Framingham pedigree totals 15 877 individuals in 1538 pedigrees consisting of both immediate and extended family. Heritability estimates were tested for significance with likelihood ratios that compared full models with reduced ones (i.e. χ^2^_1DF_ = 2 × (LogL_FULL_ − LogL_REDUCED_)) lacking the additive genetic component. Genetic correlations were also tested for significance by comparing likelihood values from full models to ones where the genetic covariance was fixed at zero.

Our genetic correlation analysis between CEB and lifespan included a total of 5133 females for whom age at death and CEB information were available. Supplementary Fig. S4 summarizes the pedigree information for these women, grouped by cohort via the ‘pedantics’ package in R [40]. Pedigree depths (computed using the same package) for the Framingham dataset range from 0 to 4, with mean 1.02 (±1.06). On average, each woman had 2.38 (±1.59) children in her lifetime and lived 77.21 (±12.73) years. The average level of education in years was 11.66. The average age at menarche was 12.81 (±1.54), average age at first birth was 26.49 (±4.81) and average age at menopause was 49.20 (±4.10).

### Genome-wide association study

Our association results are based on 444 205 SNPs from the 500 K and 50 K Affymetrix samples that satisfied the following criteria: call rate >90%, Hardy–Weinberg equilibrium *P*-value >0.00001, Mendel error rate <2% and minor allele frequency >0.01. These SNP selection criteria are further discussed in the Supplementary Information.

We used Cox proportional hazards models, as done in the phenotypic correlation analysis, to estimate the interactions between survival time past age 50 years, CEB and genotype. For censored individuals, we used their times of last observation past age 50 years as their censoring time.

Several models were run under this setup, which we number to emphasize that they are nested models. Model 1 did not adjust for any covariates. We then added covariates to reduce confounding by variables that may be correlated with lifespan and CEB. Model 2 used education level. Model 3 further added BMI, estrogen use and cohort as covariates. Models 4a–d were intermediate steps in which one of the four additional covariates was added: blood pressure treatment indicator (Model 4a), total cholesterol (Model 4b), SBP (Model 4c) and smoking indicator (Model 4d). Model 5 included all four of these additional covariates. Models 4a–d were run retrospectively to pinpoint which covariate, when added, resulted in removing significance from all SNPs. A summary of the models fitted can be found in the Supplementary Information.

Both genotypes and CEB were included as continuous variables to model an additive effect of the minor allele. We used both the raw genotypes provided by FHS as well as an imputed dataset. The imputation was done in several stages. First, we incorporated values imputed by MACH that were included in the FHS dataset. The MACH algorithm imputes missing genotypes based on shared haplotype stretches between subjects and HapMap data [41]. Of the remaining missing values, we sampled among the possible genotypes given the genotypes of parents, when parent genotypes were available. Any remaining missing values were simply sampled according to genotype proportions of the entire group. This sequence of operations created a full set of genotypes that had no missing values. Cohort was defined as a categorical variable computed from the year of birth: born before or in 1917 and born in or after 1918.

In addition to running the above five models on the full sample of 1810, we tested our models for robustness by mimicking an out-of-sample analysis. To that end, we randomly divided our sample into two equal parts and fitted Models 1–5 to each part separately to check for consistency in significance of the top performing SNPs. A true out-of-sample performance check would include the calculation of prediction error based on a model fitted on a training set. Our method does not aim to validate prediction out of sample, but rather to ensure that a SNP discovered to be significant in one sample ought to be significant in another sample—a less stringent, but still important requirement of consistency. To minimize the effects of missing genotypes on each subsample, which would further lower our sample size in each of the two separate runs, we only used the imputed genotypes for this portion of our analysis. The downside of using imputed genotypes is the risk of imputation error. To verify that our risk of imputation error is low, we used the imputed SNP data to repeat our full-sample analyses for Models 1–5. Our aim was to show that our results for these models are similar, regardless of whether we used imputed or raw SNP data.

To explore possible non-additive genotypic effects, we ran a separate Model 6 that used genotype as a categorical variable. The covariates used in Model 6 are identical to those used in Model 3, and any SNPs for which the homozygous minor genotype had fewer than 20 counts were excluded. We did not apply the half-sample testing to Model 6, because in many cases, the genotype counts in the homozygous minor allele category were too small to further subdivide the group for categorical modelling.

Finally, we ran two additional models that are outside of the nested framework given above on the raw data only (and therefore, they are not numbered). A quadratic model was run to search for a possible nonlinear effect by adding a quadratic CEB term along with its interaction with genotype to Model 1. The ‘matching covariates’ model was run to provide a frame of reference to the reader; this model uses exactly the same covariates that were included in the phenotypic and genotypic correlation analyses—education, smoking indicator and country of origin.

## RESULTS

### Phenotypic correlations

**Figure 1.**
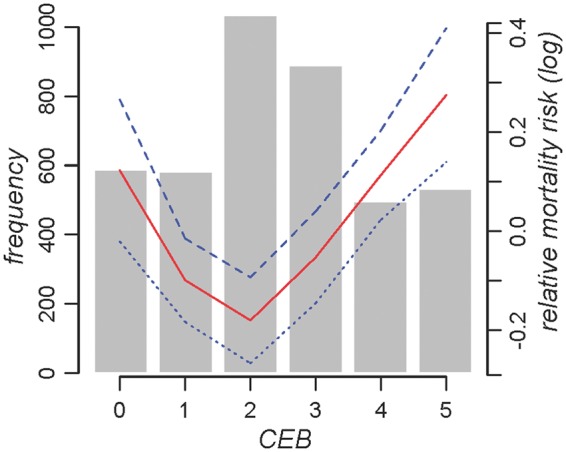
Summary of CEB and mortality risk in Framingham women. A histogram of CEB and log-relative mortality risk values for each CEB value with 95% confidence bands (*n* = 5133)

In the Cox regression analysis where as many men and women were included as possible (birth-year range 1889–1958), censoring was used to account for those who were still alive according to the latest medical records. Risk of mortality beyond age 50 years increased if women (adjusted incidence rate ratio (RR) = 1.045, *P* = 0.030) had more children (Table 1). When a nonlinear term for CEB was included, it significantly improved the model fit and became more significant than the linear term. Penalized splines for unadjusted mortality risk (Fig. 1) support a predominantly U-shaped pattern for the association between CEB and lifespan, similar to that found in some other studies (e.g. [19]). This is consistent with a cost of reproduction that is experienced by women with three or more children and with a benefit of reproduction to those who have one or two children. Highest mortality risk occurred in women with no children or more than three to four children, with lowest risk for those with approximately two. Mortality risk decreased if the first child was born later (women, unadjusted RR = 0.971, *P* < 0.001; men, adjusted RR = 0.985, *P* = 0.011; see Supplementary Fig. S1), but the significance of this effect depended on whether estimates were adjusted or not (Table 1). Mortality risk was also reduced if menopause occurred later in women (unadjusted RR = 0.970, *P* = 0.003), although this effect disappeared when other effects were controlled for (Table 1). Full model results can be seen in Supplementary Table S1.

**Table 1.** Incidence RR (±95% confidence interval) for age at death due to stroke, heart attack or cancer (beyond age 50 years)

Trait	Women		Men	
	Unadjusted	Adjusted	Unadjusted	Adjusted
CEB	1.050*	1.045*	0.995	1.031
	(1.011–1.092)^NL^**	(1.005–1.087)^NL^***	(0.960–1.033)	(0.993–1.071)
	*n* = 3729		*n* = 3888	
Age first birth	0.971***	0.977*	0.990	0.985**
	(0.955–0.988)^NL^**	(0.960–0.994)^NL^*	(0.979–1.001)	(0.974–0.995)
	*n* = 2236		*n* = 2613	
Menarche	0.891	0.917		
	(0.757–1.050)	(0.782–1.077)		
	*n* = 1367			
Menopause	0.970**	0.984		
	(0.951–0.990)	(0.965–1.005)		
	*n* = 2461			

Unadjusted Cox regression estimates included only the main predictor trait. Cultural effects (smoking, education and country-of-origin) were accounted for in adjusted estimates. ‘NL’ indicates that a significant nonlinear effect was also detected for the association between this trait and longevity. *P < 0.05, **P < 0.01, ***P < 0.001.

**Figure 2.**
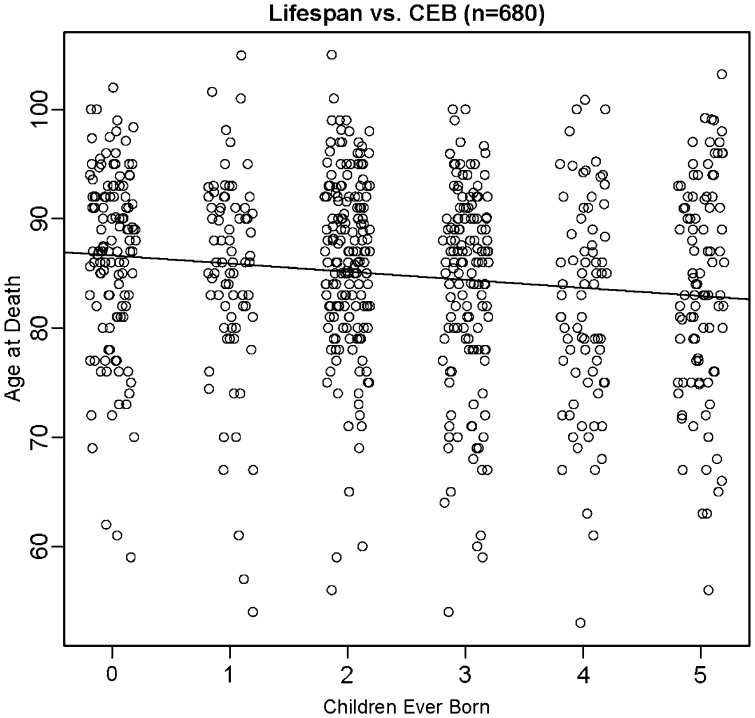
Relationship between CEB and lifespan for women. Scatterplot illustrating correlation between CEB and lifespan (*r* = −0.133, *P* < 0.001) (*n* = 680). Both variables have been jittered to minimize overlap of points

**Figure 3.**
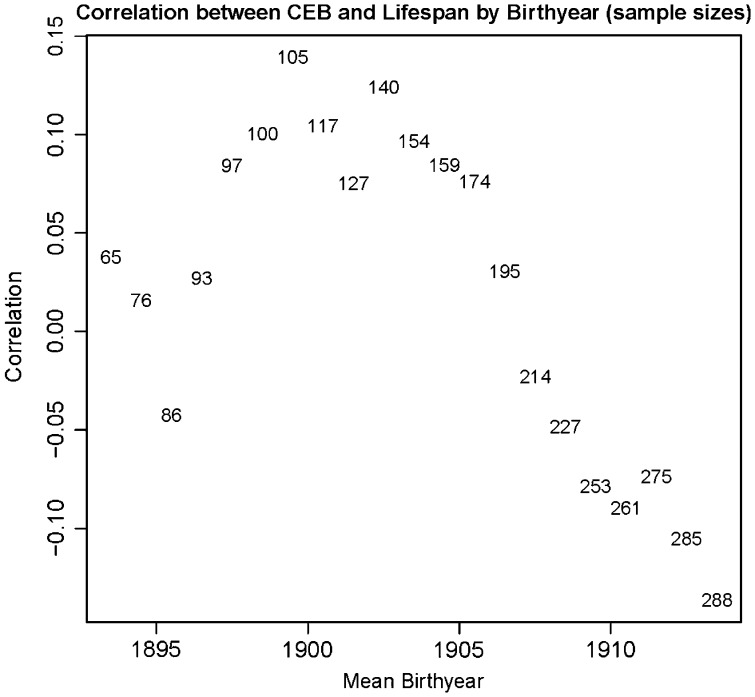
Correlation between CEB and lifespan by birth year for women. Women (*n* = 680) were grouped by overlapping 10-year intervals of birth year, and the correlation between CEB and lifespan was computed for each group. Individual points indicate the sample size of each 10-year group, with the mean birth year plotted on the *x*-axis and correlation plotted on the *y*-axis

In the analysis where only the 680 women were included in the range of birth years 1889–1918 in which all had died, the phenotypic correlation between CEB and lifespan was highly significant and negative (*r* = −0.133, *P* = 0.0005; Fig. 2). Linear regression indicated that every additional child cost 0.74 years of lifespan (standard error (SE) = 0.21 years). There was, however, significant variation in the phenotypic correlation by birth year (Fig. 3); it was positive (with one exception) from 1893 to 1907 and negative from 1908 to 1913. Many in the earlier group were giving birth before the Great Depression and World War II. Some of the latter group encountered those two major environmental perturbations. The correlation between CEB and lifespan for the 712 men was slightly negative (*r* = −0.079, *P* = 0.0355; Supplementary Fig. S2). An additional child cost 0.54 years of male lifespan (SE = 0.26 years). Again, the correlation varied by birth year, but the variations were less pronounced than for females (Supplementary Fig. S3). The observation that phenotypic correlations are dependent on birth year is consistent with previous findings that selection pressures changed over time in Framingham [38].

### Heritabilities and genetic correlations

In women (Table 2), the heritabilities of most major life-history traits differed significantly from zero, including age at death (*h*^2 ^= 0.12, *P* = 0.01), CEB (*h*^2 ^= 0.09, *P* = 0.03), age at first birth (*h*^2 ^= 0.18, *P* < 0.001) and menopause (*h*^2 ^= 0.44, *P* < 0.001).

**Table 2.** Heritabilities (*h*^2^, on the diagonal) and genetic correlations (*r*_G_, off the diagonal) of life history traits (±SE)

	Age at death	CEB	Age first birth	Menarche	Menopause
*Women*					
Age at death	**0.12 ± 0.08**	−0.69 ± 0.52	0.20 ± 0.25	0.07 ± 0.23	0.15 ± 0.17
	***P* = 0.0176**	*P* = 0.1420	*P* = 0.2083	*P* = 0.3886	*P* = 0.1917
	***n* = 3010**				
CEB		**0.09 ± 0.05**	−0.40 ± 0.35	**0.31 ± 0.24**	−0.21 ± 0.21
		***P* = 0.0394**	*P* = 0.1545	***P* < 0.0001**	*P* = 0.1377
		***n* = 4123**			
Age first birth			**0.18 ± 0.06**	−0.38 ± 0.33	−0.06 ± 0.14
			***P* = 0.0008**	*P* = 0.0911	*P* = 0.3541
			***n* = 2912**		
Menarche				0.16 ± 0.13	0.10 ± 0.21
				*P* = 0.0948	*P* = 0.3121
				*n* = 1638	
Menopause					**0.44 ± 0.06**
					***P* < 0.0001**
					*n* = 3400
*Men*					
Age at death	<0.01 ± <0.01	<0.01 ± <0.01	<0.01 ± <0.01		
	*P* = 0.8875	*P* = 0.7773	*P* = 0.6101		
	*n* = 2963				
CEB		<0.01 ± <0.01	<0.01 ± <0.01		
		*P* = 0.5485	*P* = 0.3884		
		*n* = 4051			
Age first birth			**0.12 ± 0.07**		
			***P* = 0.0300**		
			***n* = 2688**		

SEs and *P*-values were obtained from maximum-likelihood estimates. Cultural (smoking, education and country-of-origin) and maternal effects were accounted for in all estimates. *P*-values < 0.05 are in bold.

In women, the genetic correlation of CEB with age at death was large, negative and significant (*r*_G_ = −0.88, *P* = 0.01) in a model without covariates (Supplementary Table S2). When we included education as a random effect, the genetic correlation decreased to −0.70 but was still significant (*P* = 0.02). When we included either the mother or the father identifiers in place of education as a random effect, the genetic covariance remained large and negative, but was no longer significant (mother: *r*_G_ = −1.58, *P* = 0.11; father: *r*_G_ = −1.46, *P* = 0.15). The model in which we adjusted for education, smoking status and country of origin also produced a large negative genetic correlation, but the correlation was not significant (*r*_G_ = −0.69, *P* = 0.14).

The correlation between the quadratic term CEB^2^ and lifespan was large, negative and significant in three of four models (no covariates: *r*_G_ = −1.09, *P* = 0.003, only mother identifier as random effect: *r*_G_ = −1.73, *P* = 0.04, only education as random effect: *r*_G_ = −0.85, *P* = 0.01), and borderline non-significant in the model with only the father identifier (*r*_G_ = −1.61, *P* = 0.06).

Furthermore, we looked to see if the genetic correlation between CEB and lifespan was robust to pedigree depth in the simplest model where no covariates were included. Including only those women with pedigree depth of 1 or higher (*n* = 2540), we got *r*_G_ = −0.46 (*P* = 0.14) and including only those women with pedigree depth of 2 or higher (*n* = 948), we got *r*_G_ = −0.21 (*P* = 0.60); both correlations were no longer significant in the reduced samples.

The genetic correlation of CEB with age at menarche was relatively large, positive and highly significant (*r*_G_ = 0.31, *P* < 0.001). In men (Table 2), the heritability of age at first birth (inferred from their spouses) was small and only just significant (*h*^2 ^= 0.12, *P* = 0.03). All other male heritability and genetic correlation estimates were non-significant. Full model results for heritability can be seen in Supplementary Table S2.

### Genome-wide association study

GWAS results are summarized in Tables 3–10; the birth years for the 1810 women included in the GWAS are shown in the Supplementary Information. We deemed a SNP to be genome-wide significant if its interaction coefficient with CEB had a *P*-value that was less than a Bonferroni-adjusted threshold of 1.13 × 10^−^^7^ (*α* = 0.05), unless otherwise indicated. For females, we found two SNPs that attained genome-wide significance using the full sample: ss66450977 on Chromosome 3 (close to EOMES) and ss66475987 on Chromosome 4 (close to ATP8A1). Their levels of significance decreased as additional covariates were included in the model; however, these SNPs were also significant in the matching covariates model (Tables 3 and 4). We also found two nominally significant SNPs that exhibited possibly non-additive effects: ss66392234 on Chromosome 12 (in HELB) and ss66500131 on Chromosome 9 (close to the pseudogene OR7E31P) (Table 5). Nearby genes/pseudogenes were determined based on a radius of 150 kb from each SNP.

**Table 3.** GWAS for SNPs that affect the relationship between CEB and lifespan: summary of significant SNPs in Models 1–3 and 5 (full sample)

Ssid	Rsid	Chr	Position	Near	*P*-values (genotype × CEB)					
					Model 1	Model 2	Model 3	Model 4	Model 5	Matching covariates
ss66450977	rs6768456	3	27867272	EOMES	4.03E−10^a^	4.38E−10^a^	8.40E−09^a^	(see Table 4)	7.99E−07	4.93E−08^a^
ss66475987	rs2575533	4	42432336	ATP8A1	8.02E−08^a^	5.30E−08^a^	3.06E−06		2.49E−05	2.11E−07

*n* = 1810 women. The chromosome (Chr) and position information provided below correspond to the GRCh37.p5 genome assembly, genome build 37.3. ^a^SNP attained genome-wide significance.

**Table 4.** GWAS for SNPs that affect the relationship between CEB and lifespan: summary of significant SNPs in Models 4 (full sample)

Ssid	Rsid	Chr	Position	Near	*P*-values (genotype × CEB)			
					Model 4a	Model 4b	Model 4c	Model 4d
ss66450977	rs6768456	3	27867272	EOMES	1.40E−09^a^	7.44E−09^a^	8.65E−09^a^	4.02E−07
ss66475987	rs2575533	4	42432336	ATP8A1	1.02E−05	3.56E−06	5.23E−06	1.35E−05

*n* = 1810 women. The chromosome (Chr) and position information provided below correspond to the GRCh37.p5 genome assembly, genome build 37.3. ^a^SNP attained genome-wide significance.

**Table 5.** GWAS for SNPs that affect the relationship between CEB and lifespan: summary of nominally significant SNPs in Model 6

Ssid	Rsid	Chr	Position	Near	*P*-value	*P*-value	Homozygous minor genotype count
					Aa × CEB	aa × CEB	
ss66450977	rs6768456	3	27867272	EOMES	1.00E−07	2.40E−03	21
ss66500131	rs1777023	9	92008266	OR7E31P	1.00E−01	3.00E−07	26
ss66392234	rs7132724	12	65001044	HELB	1.30E−01	9.60E−08	102
ss66495977	rs2180957	14	68238574	RAD51B	1.20E−01	8.70E−07	21

*n* = 1810 women. The chromosome (Chr) and position information provided below correspond to the GRCh37.p5 genome assembly, genome build 37.3.

**Table 6.** GWAS for SNPs that affect the relationship between CEB and lifespan: re-evaluating significant SNPs in Models 1–3 and 5 (split samples)

Ssid	Sample half 1				Sample half 2			
	*P*-values (genotype × CEB)				*P*-values (genotype × CEB)			
	Model 1	Model 2	Model 3	Model 5	Model 1	Model 2	Model 3	Model 5
ss66450977	0.00032	0.00041	0.00097	0.007	9.39E−08^a^	7.04E−08^a^	1.36E−06	4.58E−06
ss66475987	0.0002	0.00012	0.0021	0.001	5.46E−04	4.46E−04	1.56E−03	1.39E−02

*n* = 1810 women. The chromosome (Chr) and position information provided below correspond to the GRCh37.p5 genome assembly, genome build 37.3. ^a^SNP attained genome-wide significance.

**Table 7.** GWAS for SNPs that affect the relationship between CEB and lifespan: re-evaluating **s**ignificant SNPs in Models 4a–d (split samples)

Ssid	Sample half 1				Sample half 2			
	*P*-values (genotype × CEB)				*P*-values (genotype × CEB)			
	Model 4a	Model 4b	Model 4c	Model 4d	Model 4a	Model 4b	Model 4c	Model 4d
ss66450977	8.40E−04	1.30E−03	8.00E−04	7.40E−03	3.33E−06	1.19E−06	1.32E−06	3.35E−06
ss66475987	3.00E−03	9.40E−04	1.80E−03	3.70E−03	2.00E−03	2.30E−03	3.80E−03	3.40E−03

*n* = 1810 women. The chromosome (Chr) and position information provided below correspond to the GRCh37.p5 genome assembly, genome build 37.3.

**Table 8.** GWAS for SNPs that affect the relationship between CEB and lifespan: top SNPs in Model 5 (split sample)

Ssid	Rsid	Chr	Position	*P*-values (genotype × CEB)	
				Sample 1	Sample 2
ss66092635	rs6581676	12	64992353	9.12E−06	4.58E−01
ss66508254	rs2961258	7	15150223	1.41E−05	7.86E−01
ss66392234	rs7132724	12	65001044	1.82E−05	4.86E−01
ss66328248	rs13248967	8	114920075	2.81E−05	6.86E−01
ss66531142	rs11219832	11	124272500	3.65E−05	1.79E−01
ss74823403	rs7860830	9	26882137	3.27E−01	7.19E−10^a^
ss66231005	rs10899741	7	52215028	4.62E−01	9.84E−08^a^
ss66273879	rs1728810	3	10992443	4.15E−01	1.07E−07^a^
ss66526690	rs1602160	6	94277193	9.00E−01	1.57E−07
ss66490007	rs11009744	10	34675601	9.86E−01	2.37E−07

*n* = 1810 women. The chromosome (Chr) and position information provided below correspond to the GRCh37.p5 genome assembly, genome build 37.3. ^a^SNP attained genome-wide significance.

**Table 9.** GWAS for SNPs that affect the relationship between CEB and lifespan: summary of significant SNPs in Models 1–3 and 5 (full sample) (imputed SNPs)

Ssid	Rsid	Chr	Position	Near	*P*-values (genotype × CEB)				
					Model 1	Model 2	Model 3	Model 4	Model 5
ss66450977	rs6768456	3	27867272	EOMES	2.91E−10^a^	2.20E−10^a^	6.44E−09^a^	(see Table 10)	5.56E−07
ss66475987	rs2575533	4	42432336	ATP8A1	1.50E−07	6.57E−08^a^	5.03E−06		2.94E−05

*n* = 1810 women. The chromosome (Chr) and position information provided below correspond to the GRCh37.p5 genome assembly, genome build 37.3. ^a^SNP attained genome-wide significance.

In the split-sample analysis using imputed SNP data (see ‘Methodology’ section regarding details on imputation), no SNPs were found to be significant for females (Tables 6–8), even when the randomization used in the split-sample assignment was replicated 100 times. We verified that using the imputed data for the full-sample analysis would have yielded comparable levels of significance for the two SNPs previously discovered in Models 1–5 (Tables 9 and 10).

**Table 10.** GWAS for SNPs that affect the relationship between CEB and lifespan: summary of significant SNPs in Model 4 (full sample) (imputed SNPs)

Ssid	Rsid	Chr	Position	Near	*P*-values (genotype × CEB)			
					Model 4a	Model 4b	Model 4c	Model 4d
ss66450977	rs6768456	3	27867272	EOMES	1.40E−08^a^	6.30E−09^a^	4.30E−09^a^	3.87E−07
ss66475987	rs2575533	4	42432336	ATP8A1	1.02E−05	5.80E−06	5.40E−06	2.30E−05

*n* = 1810 women. The chromosome (Chr) and position information provided below correspond to the GRCh37.p5 genome assembly, genome build 37.3. ^a^SNP attained genome-wide significance.

No significant SNPs were detected for males in Models 1–3. As in the GWAS for females, the addition of more covariates decreased levels of significance, and therefore no further models were run.

No significant SNPs were detected in a model that included a quadratic effect of CEB. Further details on the GWAS for females are in the Supplementary Information.

## CONCLUSIONS AND IMPLICATIONS

### Phenotypic and genetic correlations

The phenotypic correlation between CEB and lifespan in women differed with birth year, demonstrating the importance of phenotypic plasticity on the relationships among life-history traits. Secular cultural and environmental changes affect that correlation and probably account for much of the variation among studies [6, 15, 19, 21, 22]. The estimate of a negative genetic correlation in women when not accounting for covariates (*r*_G_ = −0.88) was large. The effects of shared environment reduced the strength of the linear correlation and increased the strength of the quadratic correlation, and education mimicked the effects of a cost of reproduction in that increased level of education was associated with both fewer children and longer life: including education decreased the estimate of the genetic correlation.

Some of our genetic correlation estimates were below −1. This indicates that the estimated variance component is negative, known to be a possible result of REML estimation [42].

When we controlled for the effects of smoking, education, country of origin and maternal effects, the correlation was still negative (*r*_G_ = −0.69) yet no longer significant. This mirrors the pattern we observed in the GWAS; as covariates were introduced into the model, associations became insignificant.

The mean pedigree depth of 1.02 implies that our pedigree is dominated by parent–offspring relationships. This may result in some difficulty distinguishing parental, environmental and additive genetic effects. For example, cultural and lifestyle habits that are unique to nuclear families (such as diet) are known to affect lifespan, but these habits are not recorded, and therefore the genetic correlations that we see may be confounded by these unobservable factors.

One can only find a genetic correlation when the phenotypic correlation is significant, and one can only find significant effects of SNPs on a phenotypic correlation when it differs from zero. Our chain of inference thus depends on genetic effects not being too masked by phenotypic plasticity.

### Gene functions

We found several SNPs with nominally significant effects on the correlation of CEB with post-reproductive lifespan; two of them are near EOMES and RAD51B, genes that are related to cancer when under-expressed. The effect of the SNP close to EOMES reached genome-wide significance. The EOMES gene has been associated with multiple sclerosis and bladder cancer [43, 44]. RAD51B, a gene involved in encoding proteins that participate in DNA repair, has been linked to breast cancer and brain cancer [45–48]. Further details on the genes in proximity to the SNPs found significant in our GWAS are included in the Supplementary Information. Although these SNPs were close in physical distance to their respective genes (<130 kb), further study of linkage disequilibrium would help to understand their possible association.

### Other studies

Voorhuis *et al.* [49] collated the results of many genetic studies of age at natural menopause. None of the SNPs that we discovered were found in the studies included in their summary.

Several other recent genetic studies relate fertility to genotype. Kosova *et al.* [50] found 41 SNPs (*P* < 10^−^^4^) that were associated with decreased male fertility. Adachi *et al.* [51] found 36 SNPs (*P* < 10^−^^4^) with possible links to endometriosis in Japanese females. Both were GWAS studies that did not find any genome-wide significant SNPs. Murray *et al.* [52] reported confirmations for four SNPs previously identified as associated with age at menopause. Ewens *et al.* [53] examined 15 SNPs linked with obesity to evaluate possible associations with polycystic ovary syndrome, the cause of a form of infertility in women; only one SNP had a nominal level of significance, and the significance did not hold up in another case–control study. Our methods differ fundamentally from these four studies in that we considered lifespan in conjunction with fertility, and the significant SNPs we found were not reported in their analyses [50–53].

Although the Kuningas Rotterdam study incorporated mortality in its analysis and was therefore more similar to our study [35], it differs from our approach in three ways: (i) our analysis included many more SNPs (444 205 versus their 1664), (ii) we adjusted for the effects of several direct mortality-affecting covariates such as smoking and SBP, (iii) Kuningas used an initial screening of the 1664 SNPs with a set-based test (with a threshold of *P* < 0.05), whereas we started with a GWAS across 444 205 SNPs in models that relate each SNP to both CEB and lifespan (with a threshold of *P* < 1.13 × 10^−^^7^). We did not find Bonferroni-level significance with SNPs near the four gene regions identified in [35].

### Summary

We have analysed phenotypic and genetic correlations between reproductive success and survival and have identified a small set of genes that may mediate a trade-off between them. This warrants further studies in other samples.

The Framingham dataset has some shortcomings. In particular, women born before the start of the study would only have been included in the study if they survived until 1948–52 (when the study began). Therefore, our dataset does not include anyone who died during World War I, the 1918 flu pandemic, the Great Depression and World War II. If these catastrophic events affected women differently depending on their fertility and lifespan, then excluding these women from our analysis would bias our results. The issue is inherent in such observational studies of humans, and unfortunately cannot be avoided.

We failed to find any significant SNPs when covariates (i.e. smoking, country of origin and average cholesterol levels) were included and when we did a rough check for consistency out of sample. It is unknown how often such checks modify significance of SNP associations, for many other published GWAS studies do not account for the effects of covariates or do out-of-sample predictions.

## AUTHOR CONTRIBUTIONS

S.G.B. and X.W. jointly worked on processing and cleaning the data and phenotypic correlation calculations. S.G.B. further calculated the genetic correlations and heritabilities. X.W. performed the GWAS. S.C.S. conceived of the study and drafted the initial manuscript. All authors contributed to the final manuscript.

## SUPPLEMENTARY DATA

Supplementary data is available at *EMPH* online.

Supplementary Data

## References

[1] Williams G (1957). Pleiotropy, natural selection, and the evolution of senescence. Evolution.

[2] Williams G (1966). Natural selection, costs of reproduction, and a refinement of Lack’s principle. Am Nat.

[4] Stearns SC (1992). The Evolution of Life Histories.

[5] Stearns SC, Partridge L, Masoro EJ, Austad SN (2001). The genetics of aging in Drosophila. Handbook of the Bioloy of Aging.

[6] Doblhammer G, Oeppen J (2003). Reproduction and longevity among the British peerage: the effect of frailty and health selection. Proc Biol Sci.

[7] Gagnon A, Smith KR, Tremblay M (2009). Is there a trade-off between fertility and longevity? A comparative study of women from three large historical databases accounting for mortality selection. Am J Hum Biol.

[8] Maklakov AA (2008). Sex difference in life span affected by female birth rate in modern humans. Evol Hum Behav.

[9] Tabatabaie V, Atzmon G, Rajpathak SN (2011). Exceptional longevity is associated with decreased reproduction. Aging.

[10] Thomas F, Teriokhin A, Renaud F (2000). Human longevity at the cost of reproductive success: evidence from global data. J Evol Biol.

[11] Westendorp R, Kirkwood T (1998). Human longevity at the cost of reproductive success. Nature.

[12] Fuster V (2011). Widowhood, illegitimacy, marital reproduction and female longevity in a rural Spanish population. Homo.

[13] Helle S, Lummaa V, Jokela J (2005). Are reproductive and somatic senescence coupled in humans? Late, but not early, reproduction correlated with longevity in historical sami women. Proc Biol Sci.

[14] Le Bourg E, Thon B, Légaré J (1993). Reproductive life of French–Canadians in the 17–18th centuries: a search for a trade-off between early fecundity and longevity. Exp Gerontol.

[15] McArdle P, Pollin T, O’Connell J (2006). Does having children extend life span? A genealogical study of parity and longevity in the Amish. J Gerontol Ser A Biol Sci Med Sci.

[16] Muller HG, Chiou JM, Carey JR (2002). Fertility and life span: late children enhance female longevity. J Gerontol Ser A Biol Sci Med Sci.

[17] Sear R (2007). The impact of reproduction on Gambian women: does controlling for phenotypic quality reveal costs of reproduction?. Am J Phys Anthropol.

[18] Lawlor DA, Emberson JR, Ebrahim S (2003). Is the association between parity and coronary heart disease due to biological effects of pregnancy or adverse lifestyle risk factors associated with child-rearing? Findings from the British Women’ Heart and Health Study and the British Regional Heart Study. Circulation.

[19] Lund EE, Arnesen EE, Borgan JKJ (1990). Pattern of childbearing and mortality in married women—a national prospective study from Norway. J Epidemiol Commun Health.

[20] Manor OO, Eisenbach ZZ, Israeli AA Mortality differentials among women: the Israel Longitudinal Mortality Study. Soc Sci Med (1967).

[21] Dribe M (2004). Long-term effects of childbearing on mortality: evidence from pre-industrial Sweden. Popul Stud.

[22] Gavrilova N, Gavrilov L, Semyonova VG (2004). Does exceptional human longevity come with a high cost of infertility? Testing the evolutionary theories of aging. Ann N Y Acad Sci.

[23] Helle S, Käär P, Jokela J (2002). Human longevity and early reproduction in pre-industrial Sami populations. J Evol Biol.

[24] Jacobsen BK, Knutsen SF, Oda K (2011). Parity and total, ischemic heart disease and stroke mortality. The Adventist Health Study, 1976–1988. Eur J Epidemiol.

[25] Jasienska G, Nenko I, Jasienski M (2006). Daughters increase longevity of fathers, but daughters and sons equally reduce longevity of mothers. Am J Hum Biol.

[26] Korpelainen H (2000). Fitness, reproduction and longevity among European aristocratic and rural Finnish families in the 1700s and 1800s. Proc Biol Sci.

[27] Lycett JE, Dunbar RIM, Voland E (2000). Longevity and the costs of reproduction in a historical human population. Proc Biol Sci.

[28] Cesarini D, Lindqvist E, Wallace B (2007). Maternal longevity and the sex of offspring in pre-industrial Sweden. Ann Hum Biol.

[29] Gavrilov L, Gavrilova N (1999). Is there a reproductive cost for human longevity?. J Anti-Aging Med.

[30] Jasienska G (2009). Reproduction and lifespan: trade-offs, overall energy budgets, intergenerational costs, and costs neglected by research. Am J Hum Biol.

[31] Mitteldorf J (2010). Female fertility and longevity. Age.

[32] Le Bourg E (2007). Does reproduction decrease longevity in human beings?. Ageing Res Rev.

[33] Stearns S, Ackermann M, Doebeli M (2000). Experimental evolution of aging, growth, and reproduction in fruitflies. Proc Natl Acad Sci USA.

[34] Gögele M, Pattaro C, Fuchsberger C (2011). Heritability analysis of life span in a semi-isolated population followed across four centuries reveals the presence of pleiotropy between life span and reproduction. J Gerontol Ser A Biol Sci Med Sci.

[35] Kuningas M, Altmäe S, Uitterlinden AG (2011). The relationship between fertility and lifespan in humans. Age.

[36] R Core Team (2009). R: A Language and Environment for Statistical Computing.

[37] http://CRAN.R-project.org/package=survival.

[38] Byars SG, Ewbank D, Govindaraju DR (2010). Colloquium papers: natural selection in a contemporary human population. Proc Natl Acad Sci.

[39] Gilmour A, Gogel B, Cullis B (2002). ASReml user guide release 3.0.

[40] Morrissey MB, Wilson A (2010). Pedantics: an R package for pedigree-based genetic simulation and pedigree manipulation, characterization and viewing. Molecular Ecology Resources.

[41] Li Y, Abecasis GR (2006). Mach 1.0: rapid haplotype reconstruction and missing genotype inference. Am J Hum Genet.

[42] Thompson WA (1962). The problem of negative estimates of variance components. Ann Math Stat.

[43] Reinert T, Modin C, Castano FM (2011). Comprehensive genome methylation analysis in bladder cancer: identification and validation of novel methylated genes and application of these as urinary tumor markers. Clin Cancer Res.

[44] Patsopoulos NA, de Bakker PIW (2011). Genome-wide meta-analysis identifies novel multiple sclerosis susceptibility loci. Ann Neurol.

[45] Liu Y, Shete S, Wang LE (2010). Gamma-radiation sensitivity and polymorphisms in RAD51L1 modulate glioma risk. Carcinogenesis.

[46] Figueroa JD, Garcia-Closas M, Humphreys M (2011). Associations of common variants at 1p11. 2 and 14q24. 1 (RAD51L1) with breast cancer risk and heterogeneity by tumor subtype: findings from the Breast Cancer Association Consortium. Hum Mol Genet.

[47] Shu XO, Long J, Lu W (2012). Novel genetic markers of breast cancer survival identified by a genome-wide association study. Cancer Res.

[48] Wibom C, Sjöström S, Henriksson R (2012). DNA-repair gene variants are associated with glioblastoma survival. Acta Oncol.

[49] Voorhuis M, Onland-Moret NC, van der Schouw YT (2010). Human studies on genetics of the age at natural menopause: a systematic review. Hum Reprod Update.

[50] Kosova G, Scott NM, Niederberger C (2012). Genome-wide association study identifies candidate genes for male fertility traits in humans. Am J Hum Genet.

[51] Adachi S, Tajima A, Quan J (2010). Meta-analysis of genome-wide association scans for genetic susceptibility to endometriosis in Japanese population. J Hum Genet.

[52] Murray A, Bennett CE, Perry JRB (2010). Common genetic variants are significant risk factors for early menopause: results from the Breakthrough Generations Study. Hum Mol Genet.

[53] Ewens KG, Jones MR, Ankener W (2011). FTO and MC4R gene variants are associated with obesity in polycystic ovary syndrome. PLoS One.

