# Supplementary material for: Genetic links between post-reproductive lifespan and family size in Framingham
Source: Evol Med Public Health. 2013 Jun 25;2013(1):241–53. doi: 10.1093/emph/eot013 (PMC3868361; doi:10.1093/emph/eot013)
Supplement: Supplementary Data [file supp_2013_1_241__index.html]

Genetic links between post-reproductive lifespan and family size in Framingham — Genetic links between post-reproductive lifespan and family size in Framingham — Supplementary Data 

# Genetic links between post-reproductive lifespan and family size in Framingham

## 

files

**Files in this Data Supplement:**

- Supplementary Data - docx file
- Supplementary Data - tiff file
- Supplementary Data - tif file
- Supplementary Data - tif file
- Supplementary Data - tif file
- Supplementary Data - docx file
