# Supplementary material for: Genetic links between post-reproductive lifespan and family size in Framingham
Source: Evol Med Public Health. 2013 Jun 25;2013(1):241–53. doi: 10.1093/emph/eot013 (PMC3868361; doi:10.1093/emph/eot013)
Supplement: Supplementary Data [file supp_eot013_EMPH_supplementary.docx]

**Supplementary Information**

## SNP Exclusion Criteria

We excluded SNPs that failed to attain one of the following conditions, for reasons that are explained below:

1. Call rate > 90% - the proportion of SNPs that could be classified as either ‘AA’ (0), ‘Aa’ (1), or ‘aa’ (2), where ‘A’ is the major allele and ‘a’ is the minor allele. A low call rate may imply the inherent difficulty in separating out the various genotypes.
2. Hardy-Weinberg Equilibrium p-value > 0.00001 – A low Hardy-Weinberg Equilibrium p-value suggests a possible genotyping error.
3. Mendel error rate < 2% - Mendel error rate is the proportion of inconsistent genotypes. For example, parent genotypes of (AA x AA) with an associated child genotype of aa would be a Mendel error.
4. Minor allele frequency > 0.01 – SNPs with low minor allele frequencies are more vulnerable to genotyping error, and are thus excluded.

References:

Pongpanich, Monnat, Patrick F. Sullivan, and Jung-Ying Tzeng. "A quality control algorithm for filtering SNPs in genome-wide association studies."Bioinformatics 26.14 (2010): 1731-1737.

## Cox, David G., and Peter Kraft. "Quantification of the power of Hardy-Weinberg equilibrium testing to detect genotyping error." Human Heredity 61.1 (2006): 10-14.

## GWAS Models

| **Model** | **Details** |
| --- | --- |
| 1 | $\ln\left( \frac{H(t)}{{H(t}_{0})} \right)=\beta_{1}\times CEB+\beta_{2}\times SNP+\beta_{3}\times CEB\times SNP$ |
| 2 | $\ln\left( \frac{H(t)}{{H(t}_{0})} \right)=\beta_{1}\times CEB+\beta_{2}\times SNP+\beta_{3}\times CEB\times SNP+\beta_{4}\times Education$ |
| 3 | $\ln\left( \frac{H(t)}{{H(t}_{0})} \right)=\beta_{1}\times CEB+\beta_{2}\times SNP+\beta_{3}\times CEB\times SNP+\beta_{4}\times BMI+\beta_{5}\times Estrogen use+\beta_{6}\times Cohort+\beta_{7}\times Education$ |
| 4a | $\ln\left( \frac{H\left( t \right)}{{H(t}_{0})} \right)=\beta_{1}\times CEB+\beta_{2}\times SNP+\beta_{3}\times CEB\times SNP+\beta_{4}\times BMI+\beta_{5}\times Estrogen use+\beta_{6}\times Cohort+\beta_{7}\times Education+\beta_{8}\times Blood Pressure Treatment Flag$ |
| 4b | $\ln\left( \frac{H\left( t \right)}{{H(t}_{0})} \right)=\beta_{1}\times CEB+\beta_{2}\times SNP+\beta_{3}\times CEB\times SNP+\beta_{4}\times BMI+\beta_{5}\times Estrogen use+\beta_{6}\times Cohort+\beta_{7}\times Education+\beta_{8}\times Total Cholesterol$ |
| 4c | $\ln\left( \frac{H\left( t \right)}{{H(t}_{0})} \right)=\beta_{1}\times CEB+\beta_{2}\times SNP+\beta_{3}\times CEB\times SNP+\beta_{4}\times BMI+\beta_{5}\times Estrogen use+\beta_{6}\times Cohort+\beta_{7}\times Education+\beta_{8}\times Systolic Blood Pressure$ |
| 4d | $\ln\left( \frac{H\left( t \right)}{{H(t}_{0})} \right)=\beta_{1}\times CEB+\beta_{2}\times SNP+\beta_{3}\times CEB\times SNP+\beta_{4}\times BMI+\beta_{5}\times Estrogen use+\beta_{6}\times Cohort+\beta_{7}\times Education+\beta_{8}\times Smoking Flag$ |
| 5 | $\ln\left( \frac{H\left( t \right)}{{H(t}_{0})} \right)=\beta_{1}\times CEB+\beta_{2}\times SNP+\beta_{3}\times CEB\times SNP+\beta_{4}\times BMI+\beta_{5}\times Estrogen use+\beta_{6}\times Cohort+\beta_{7}\times Education+\beta_{8}\times Blood Pressure Treatment Flag+\beta_{9}\times Total Cholesterol+\beta_{10}\times Systolic Blood Pressure+\beta_{11}\times Smoking Flag$ |
| Quadratic | $\ln\left( \frac{H(t)}{{H(t}_{0})} \right)=\beta_{1}\times CEB+\beta_{2}\times SNP+\beta_{3}\times CEB\times SNP+\beta_{4}\times{CEB}^{2}+\beta_{5}\times{CEB}^{2}\times SNP$ |
| Same  Covariates | $\ln\left( \frac{H\left( t \right)}{{H(t}_{0})} \right)=\beta_{1}\times CEB+\beta_{2}\times SNP+\beta_{3}\times CEB\times SNP+\beta_{4}\times Education+\beta_{5}\times Country of Origin+\beta_{6}\times Smoking Flag$ |

**Frequency of birth years for GWAS dataset (n = 1810 women)**

| **Birth year count** | | | | | | | | | | | | | | | |
| --- | --- | --- | --- | --- | --- | --- | --- | --- | --- | --- | --- | --- | --- | --- | --- |
| **1889** | **1890** | **1891** | **1892** | **1893** | **1894** | **1895** | **1896** | **1897** | **1898** | **1899** | **1900** | **1901** | **1902** | **1903** | **1904** |
| 1 | 2 | 5 | 4 | 9 | 8 | 9 | 10 | 2 | 15 | 12 | 12 | 12 | 8 | 12 | 13 |
| **1905** | **1906** | **1907** | **1908** | **1909** | **1910** | **1911** | **1912** | **1913** | **1914** | **1915** | **1916** | **1917** | **1918** | **1919** | **1920** |
| 22 | 20 | 18 | 32 | 21 | 38 | 38 | 38 | 35 | 55 | 54 | 50 | 54 | 66 | 61 | 51 |
| **1921** | **1922** | **1923** | **1924** | **1925** | **1926** | **1927** | **1928** | **1929** | **1930** | **1931** | **1932** | **1933** | **1934** | **1935** | **1936** |
| 30 | 21 | 39 | 51 | 35 | 44 | 59 | 48 | 52 | 60 | 51 | 70 | 57 | 56 | 48 | 51 |
| **1937** | **1938** | **1939** | **1940** |  |  |  |  |  |  |  |  |  |  |  |  |
| 57 | 64 | 57 | 73 |  |  |  |  |  |  |  |  |  |  |  |  |

**GWAS Result Details for Women**

On the full sample (n = 1,810), we found 2 significant SNP interactions in Model 1, where no covariates were used (Table 3a). The correlation between the two SNPs was close to zero; they appear to be independent. One of the SNPs (ss66450977 on Chromosome 3, close to EOMES) remained significant with the addition of covariates in Models 2 and 3 (and Models 4a-c). The other SNP (ss66475987 on Chromosome 4, close to ATP8A1) was also significant in Model 2. No SNPs reached genome-wide significance in Model 5, although ss66450977 attained an interaction p-value of 7.99×10^-7^. Our results from Models 4a-d (Table 3b) suggest that the addition of the smoking indicator (Model 4d) resulted in our loss of significance, as the other three models still showed the EOMES SNP to be genome-wide significant.

In our non-additive Model 6, with education level, BMI, estrogen use, and cohort (before or after 1917) as covariates, no SNPs were genome-wide significant, but four SNPs had effects on the relationship between CEB and lifespan that were nominally significant, under the criterion that one of the two interaction coefficients had a p-value smaller than 1e-6 (Table 4c). The pairwise correlations of the four SNPs were again close to zero.

In the next part of our analysis, we split the sample in half to fit the same type of model (Models 1-5) to either half. Doing so resulted in zero SNPs that consistently reached the Bonferroni threshold in both samples. Our previously-found significant SNPs at most had a competitive p-value in only one of the two models (Tables 3d-e).

We took a deeper look at the split runs for Model 5. One of the two samples produced 0 SNPs that attained the Bonferroni threshold, while the other produced 3 SNPs that did. Table 2f summarizes the top 5 SNPs in each sample. Note that one SNP previously identified in Model 6 (ss66392234 on Chromosome 12, in HELB) was also found on the top 5 list for Sample 1. As can be seen, there is no overlap in the top performers. In fact, the 3 SNPs in Sample 2 that did achieve genome-wide significance had p-values larger than 0.3 in Sample 1.

To verify that the lack of consistently significant SNPs in these split-sample models was not due to chance, we replicated our analyses on 100 random splits of the data. Out of these 100 random partitions, we still failed to discover a single one in which a SNP showed significance in both models fit to each half of the data.

Furthermore, Tables 2g-h show that using the imputed SNP data yielded similar results on our full sample Models 1-5. Once again, we found the EOMES SNP to be significant in Models 1, 2, 3, and 4a-c, but falling short in Model 5. This shows that our usage of imputed SNP data for the split-sample analysis should not have significantly hurt our significance levels.

## Known Information About Discovered SNPs

| The chromosome (Chr) and position information provided below correspond to the GRCh37.p5 genome assembly, genome build 37.3. |  |  |  |  |
| --- | --- | --- | --- | --- |

| **Ssid / rsid** | **Chr** | **Position** | **Nearby Gene/Pseudo-gene**  **(distance)** | **Gene/Pseudo-gene Details** |
| --- | --- | --- | --- | --- |
| ss66450977 / rs6768456 | 3 | 27,892,268 | EOMES (130Kbp) | EOMES encodes a member of a protein family that shares a common DNA-binding domain, the T-box, whose genes encode transcription factors involved in the regulation of developmental processes.  [Genome-wide meta-analysis identifies novel multiple sclerosis susceptibility loci.](http://www.ncbi.nlm.nih.gov/pubmed/22190364/) Patsopoulos NA, *et al*. Ann Neurol, 2011 Dec. PMID 22190364.  *SNP rs170934 near the gene was discovered as a novel susceptibility allele for multiple sclerosis in a meta-analysis.*  Reinert, Thomas, et al. "Comprehensive genome methylation analysis in bladder cancer: identification and validation of novel methylated genes and application of these as urinary tumor markers." Clinical Cancer Research 17.17 (2011): 5582-5592.  *The EOMES gene was found to be a novel methylation tumor marker for the early detection of bladder cancer.*  Zhu, Yibei, et al. "T-bet and eomesodermin are required for T cell-mediated antitumor immune responses." *The Journal of Immunology* 185.6 (2010): 3174-3183.  *The EOMES gene was shown to play a role in immune responses against tumors in mice.* |
| ss66500131 / rs1777023 | 9 | 92,968,446 | OR7E31P and  OR7E116P  (< 40 Kbp) | Both are olfactory receptors pseudogenes. |
| ss66392234 / rs7132724 | 12 | 66,714,777 | HELB  (0 Kbp, intron SNP) | Also known as human DNA helicase B, or HDHB, this gene encodes a protein that is involved in the unwinding of DNA for repair, replication, recombination, and transcription.  Human DNA helicase B (HDHB) binds to replication protein A and facilitates cellular recovery from replication stress. Guler GD, et al. J Biol Chem, 2012 Feb 24. PMID 22194613.  A dominant-negative mutant of human DNA helicase B blocks the onset of chromosomal DNA replication. Taneja P, et al. J Biol Chem, 2002 Oct 25. PMID 12181327. |
| ss66475987 / rs2575533 | 4 | 42,737,579 | ATP8A1  (100 Kbp) | Also known as ATPIA, ATPP2, and ATPASEII, this gene encodes an ATPase-family protein which transports amphipaths, such as phosphatidylserine.  Levano, Kelly, et al. "Atp8a1 deficiency is associated with phosphatidylserine externalization in hippocampus and delayed hippocampus‐dependent learning." Journal of neurochemistry (2012).  Genome-wide analysis of polymorphisms associated with cytokine responses in smallpox vaccine recipients. Kennedy RB, et al. Hum Genet, 2012 Sep. PMID 22610502.  *3 SNPs in the gene have suggestive association with changes in cytokine response to smallpox vaccines.* |
| ss66495977 / rs2180957 | 14 | 69,168,821 | RAD51B  (110 Kbp) | Also known as REC2, R51H2, and RAD51L1, this gene encodes a protein in the RAD51 family, which aid in DNA repair. Overexpression of the gene has been shown to result in cell cycle G1 delay and cell apoptosis. Much research has shown that the gene is associated with cancer.  Novel genetic markers of breast cancer survival identified by a genome-wide association study. Shu XO, et al. Cancer Res, 2012 Mar 1. PMID 22232737.  *SNP rs3784099 in the gene was one of two SNPs identified in a two-stage GWAS for association with total mortality in breast cancer.*  Associations of common variants at 1p11.2 and 14q24.1 (RAD51L1) with breast cancer risk and heterogeneity by tumor subtype: findings from the Breast Cancer Association Consortium. Figueroa JD, et al. Hum Mol Genet, 2011 Dec 1. PMID 21852249.  *Two representative SNPs for the gene were examined in a case-control study of various breast cancer tumor types. Both demonstrated an association with breast cancer. The study further quantifies risk estimates for different types of tumor characteristics.*  Gamma-radiation sensitivity and polymorphisms in RAD51L1 modulate glioma risk. Liu Y, Shete S, Wang LE, El-Zein R, Etzel CJ, Liang FW, Armstrong G, Tsavachidis S, Gilbert MR, Aldape KD, Xing J, Wu X, Wei Q, Bondy ML.  Carcinogenesis. 2010 Oct;31(10):1762-9. Epub 2010 Jul 7.  *SNP rs2180611 was found to be significantly associated with mutagen sensitivity. The results suggest that the gene modulates glioma risk.*  DNA-repair gene variants are associated with glioblastoma survival.  Wibom C, Sjöström S, Henriksson R, Brännström T, Broholm H, Rydén P, Johansen C, Collatz-Laier H, Hepworth S, McKinney PA, Bethke L, Houlston RS, Andersson U, Melin BS. Acta Oncol. 2012 Mar ;51(3):325-32. Epub 2011 Oct 21.  *Five SNPs in the gene were significantly associated with glioblastoma survival.*  Comprehensive pathway-based association study of DNA repair gene variants and the risk of nasopharyngeal carcinoma. Qin HD, Shugart YY, Bei JX, Pan QH, Chen L, Feng QS, Chen LZ, Huang W, Liu JJ, Jorgensen TJ, Zeng YX, Jia WH. Cancer Res. 2011 Apr 15;71(8):3000-8. Epub 2011 Mar 2.  *Two SNPs in the gene were found to be strongly associated with nasopharyngeal carcinoma in Cantonese population study.* |

**Supplementary Figures**

**Figure S1 – Summary of Age at First Birth (AFB) and mortality risk in Framingham women**

A histogram of AFB and log-relative mortality risk values for mother’s age at first birth with 95% confidence bands (n = 5,133).

**Figure S2 – Relationship between CEB and lifespan for men**

Scatterplot illustrating correlation between CEB and lifespan (r = -0.079, p-value = 0.036) (n = 712). Both variables have been jittered to minimize overlap of points.

**Figure S3 – Correlation between CEB and lifespan by birth year for men**

Men (n = 712) were grouped by overlapping 10-year intervals of birth year, and the correlation between CEB and lifespan was computed for each group. Individual points indicate the sample size of each 10-year group, with the mean birth year plotted on the *x*-axis and correlation plotted on the *y*-axis.

**Figure S4 – Pedigree of informative females separated by cohort**

Pedigree plot displaying the n = 5,133 individuals used in the CEB-lifespan genetic correlation analysis, grouped by cohort. A cohort value of -1 indicates the founders of the pedigrees. Mean pedigree depth for these individuals was calculated to be 1.02 (± 1.06).
