# Supplementary figures and images for: Genetic links between post-reproductive lifespan and family size in Framingham
Source: Evol Med Public Health. 2013 Jun 25;2013(1):241–53. doi: 10.1093/emph/eot013 (PMC3868361; doi:10.1093/emph/eot013)

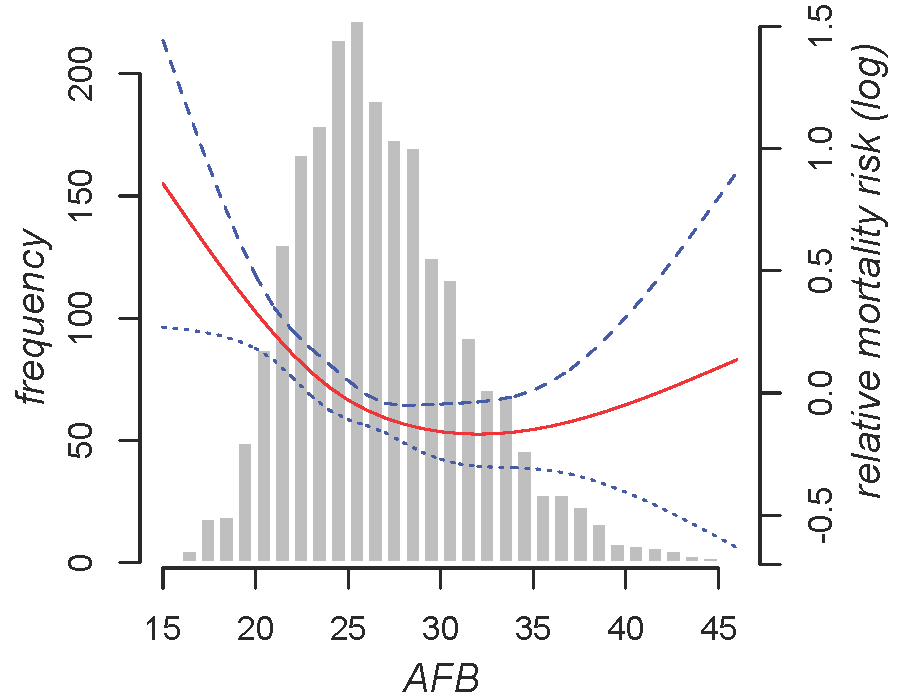

Supplement: Supplementary Data [file supp_eot013_S1_supplementary.tif]

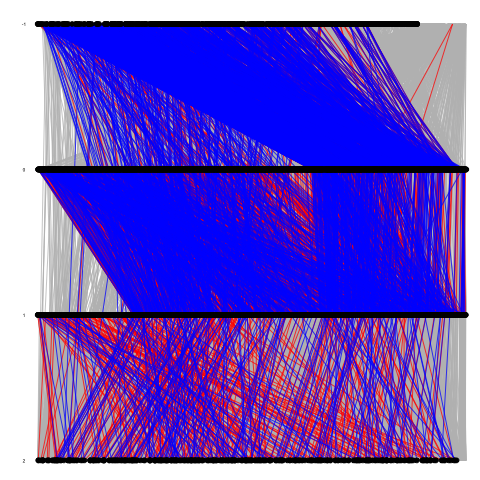

Supplement: Supplementary Data [file supp_eot013_S4_supplementary.tif]
