# Supplementary material for: Genetic links between post-reproductive lifespan and family size in Framingham
Source: Evol Med Public Health. 2013 Jun 25;2013(1):241–53. doi: 10.1093/emph/eot013 (PMC3868361; doi:10.1093/emph/eot013)
Supplement: Supplementary Data [file supp_eot013_Supplementary_tables.docx]

# Table S1 - Incidence rate ratio (± 95% confidence interval) for age at death due to stroke, heart attack or cancer (beyond age 50) – full outputs. CEB (children ever born), AFB (age at first birth), Menar (age at menopause), Meno (age at menopause).

|  | **women** | **men** |
| --- | --- | --- |
|  | **RR (± 95% c.i.)** | **RR (± 95% c.i.)** |
| **CEB** (unadjusted) | 1.050 (1.011–1.092)* | 0.995 (0.960-1.033) |
| **CEB** (adjusted) | 1.045 (1.005-1.087)* | 1.031 (0.993-1.071) |
| smoking | 1.270 (1.116-1.446)*** | 1.515 (1.313-1.748)*** |
| education | 0.974 (0.954-0.996)* | 0.964 (0.949-0.979)*** |
| country | 1.091 (0.917-1.299) | 0.903 (0.776-1.052) |
|  |  |  |
| **AFB** (unadjusted) | 0.971 (0.955-0.988)*** | 0.990 (0.979- 1.001) |
| **AFB** (adjusted) | 0.977 (0.960-0.994)* | 0.985 (0.974-0.995)** |
| smoking | 1.395 (1.170-1.664)*** | 1.481 (1.255-1.748)*** |
| education | 0.972 (0.944-1.002) | 0.966 (0.948-0.985)*** |
| country | 1.372 (1.093-1.723)** | 0.937 (0.785-1.118) |
|  |  |  |
| **Menar** (unadjusted) | 0.891 (0.757- 1.050) |  |
| **Menar** (adjusted) | 0.917 (0.782-1.077) |  |
| smoking | 1.841 (1.115-3.040)* |  |
| education | 0.980 (0.886-1.084) |  |
| country | - |  |
|  |  |  |
| **Meno** (unadjusted) | 0.970 (0.951- 0.990)** |  |
| **Meno** (adjusted) | 0.984 (0.965-1.005) |  |
| smoking | 1.364 (1.160-1.605)*** |  |
| education | 0.965 (0.941-0.990)** |  |
| country | 1.141 (0.926-1.407) |  |
|  |  |  |

# Table S2 - Heritabilities (*h^2^*, on the diagonal), genetic correlations (*r_G_*, off the diagonal) and other variance components of life history traits (± SE). First row for each trait represents unadjusted estimates. Second row represents adjusted (for cultural and maternal covariates) estimates. Remaining rows are variance components or *P*-values for random (maternal effects, education) and fixed (country-of-origin, smoking) effects, respectively. Standard errors and *P*-values for variance components were obtained from maximum likelihood estimates. *P*-values for fixed effects were obtained from Analysis of Variance (ANOVA). CEB (children ever born), AFB (age at first birth), Menar (age at menopause), Meno (age at menopause).

## *(a) Women*

|  | **Age at Death** | **CEB** | **AFB** | **Menar** | **Meno** |  |
| --- | --- | --- | --- | --- | --- | --- |
| **Age at Death** | **0.20 ± 0.08** | **-0.88 ± 0.41** | 0.20 ± 0.25 | 0.07 ± 0.23 | 0.15 ± 0.17 | unadjusted |
| **Age at Death** | **0.12 ± 0.08** | **-0.69 ± 0.52** | 0.20 ± 0.25 | 0.07 ± 0.23 | 0.15 ± 0.17 | adjusted |
|  | **0.14 ± 0.04** | -0.46 ± 0.47 | <0.01±<0.01 | 0.03 ± 0.58 | <0.01±<0.01 | maternal |
|  | 0.01 ± 0.01 | -0.27 ± 0.47 | 0.75 ± 0.23 | 0.66 ± 0.40 | 0.81 ± 0.20 | education |
|  | **< 0.001** | **< 0.001** | **< 0.001** | **< 0.001** | **< 0.001** | country |
|  | **< 0.001** | **< 0.001** | **< 0.001** | **< 0.001** | **< 0.001** | smoking |
|  |  |  |  |  |  |  |
| **CEB** |  | **0.09 ± 0.05** | **-0.66 ± 0.26** | **0.64 ± 0.32** | -0.21 ± 0.21 | unadjusted |
| **CEB** |  | **0.09 ± 0.05** | -0.40 ± 0.35 | **0.31 ± 0.24** | -0.21 ± 0.21 | adjusted |
|  |  | 0.04 **±** 0.03 | <0.01±<0.01 | -0.01 ± 0.55 | 0.11 ± 0.04 | maternal |
|  |  | 0.06 **±** 0.03 | -0.95 ± 0.04 | -0.13 ± 0.75 | <0.01±<0.01 | education |
|  |  | 0.106 | **0.042** | 0.105 | **<0.001** | country |
|  |  | **0.007** | **< 0.001** | **0.037** | **0.001** | smoking |
|  |  |  |  |  |  |  |
| **AFB** |  |  | **0.22 ± 0.06** | -0.37 ± 0.21 | -0.14 ± 0.14 | unadjusted |
| **AFB** |  |  | **0.18 ± 0.06** | -0.38 ± 0.33 | -0.06 ± 0.14 | adjusted |
|  |  |  | <0.01±<0.01 | <0.01±<0.01 | <0.01±<0.01 | maternal |
|  |  |  | 0.10 ± 0.05 | 0.48 ± 0.54 | 0.90 ± 0.09 | education |
|  |  |  | 0.072 | 0.077 | **0.012** | country |
|  |  |  | **< 0.001** | **< 0.001** | **< 0.001** | smoking |
|  |  |  |  |  |  |  |
| **Menar** |  |  |  | **0.37 ± 0.10** | 0.10 ± 0.14 | unadjusted |
| **Menar** |  |  |  | 0.16 ± 0.13 | 0.10 ± 0.21 | adjusted |
|  |  |  |  | **0.13 ± 0.06** | 0.47 ± 0.89 | maternal |
|  |  |  |  | 0.01 **±** 0.01 | 0.19 ± 0.61 | education |
|  |  |  |  | - | **0.004** | country |
|  |  |  |  | 0.823 | **< 0.001** | smoking |
|  |  |  |  |  |  |  |
| **Meno** |  |  |  |  | **0.48 ± 0.06** | unadjusted |
| **Meno** |  |  |  |  | **0.44 ± 0.06** | adjusted |
|  |  |  |  |  | 0.02 ± 0.04 | maternal |
|  |  |  |  |  | 0.03 ± 0.02 | education |
|  |  |  |  |  | **0.004** | country |
|  |  |  |  |  | **< 0.001** | smoking |

## *(b) Men*

|  | **Age at Death** | **CEB** | **AFB** |  |
| --- | --- | --- | --- | --- |
| **Age at Death** | 0.12 ± 0.07 | <0.01 ± <0.01 | 0.22 ± 0.44 | unadjusted |
| **Age at Death** | <0.01 ± <0.01 | <0.01 ± <0.01 | <0.01 ± <0.01 | adjusted |
|  | **0.22 ± 0.04** | **0.79 ± 0.15** | <0.01 ± <0.01 | maternal |
|  | 0.01 ± 0.01 | <0.01±<0.01 | -0.01 ± 0.69 | education |
|  | **< 0.001** | **< 0.001** | **< 0.001** | country |
|  | 0.137 | 0.424 | **0.006** | smoking |
|  |  |  |  |  |
| **CEB** |  | <0.01 ± <0.01 | <0.01 ± <0.01 | unadjusted |
| **CEB** |  | <0.01 ± <0.01 | <0.01 ± <0.01 | adjusted |
|  |  | **0.20 ± 0.03** | -0.88 ± 0.95 | maternal |
|  |  | 0.01 ± 0.01 | 0.43 ± 0.53 | education |
|  |  | **0.002** | **< 0.001** | country |
|  |  | 0.964 | 0.136 | smoking |
|  |  |  |  |  |
| **AFB** |  |  | **0.11 ± 0.06** | unadjusted |
| **AFB** |  |  | **0.12 ± 0.07** | adjusted |
|  |  |  | 0.01 ± 0.04 | maternal |
|  |  |  | <0.01 ± <0.01 | education |
|  |  |  | **< 0.001** | country |
|  |  |  | **0.018** | smoking |
